# Elucidating the Role of Effectors in Plant-Fungal Interactions: Progress and Challenges

**DOI:** 10.3389/fmicb.2016.00600

**Published:** 2016-04-27

**Authors:** Carrie Selin, Teresa R. de Kievit, Mark F. Belmonte, W. G. Dilantha Fernando

**Affiliations:** ^1^Department of Plant Science, University of ManitobaWinnipeg, MB, Canada; ^2^Department of Microbiology, University of ManitobaWinnipeg, MB, Canada; ^3^Department of Biological Sciences, University of ManitobaWinnipeg, MB, Canada

**Keywords:** effectors, biotrophs, necrotrophs, hemibiotrophs, plant pathogen interactions, plant resistance

## Abstract

Pathogenic fungi have diverse growth lifestyles that support fungal colonization on plants. Successful colonization and infection for all lifestyles depends upon the ability to modify living host plants to sequester the necessary nutrients required for growth and reproduction. Secretion of virulence determinants referred to as “effectors” is assumed to be the key governing factor that determines host infection and colonization. Effector proteins are capable of suppressing plant defense responses and alter plant physiology to accommodate fungal invaders. This review focuses on effector molecules of biotrophic and hemibiotrophic plant pathogenic fungi, and the mechanism required for the release and uptake of effector molecules by the fungi and plant cells, respectively. We also place emphasis on the discovery of effectors, difficulties associated with predicting the effector repertoire, and fungal genomic features that have helped promote effector diversity leading to fungal evolution. We discuss the role of specific effectors found in biotrophic and hemibiotrophic fungi and examine how CRISPR/Cas9 technology may provide a new avenue for accelerating our ability in the discovery of fungal effector function.

## Introduction

Fungal plant pathogens are economically important due to the threat they pose to the production and yield of agricultural crops. It has been estimated that global agricultural production suffers an average annual loss of 15% due to plant diseases (Oerke, 2006; Lo Presti et al., 2015; Schwessinger et al., 2015). To reduce and/or prevent fungal plant diseases, farmers depend on resistant crop cultivars or fungicide treatments. Furthermore, current agricultural cropping strategies rely primarily on the rotation of one cropping genotype within large areas of land, promoting the selection of fungal isolates capable of overcoming crop resistance. Such farming practices impose the need for continuous development and introduction of new genetic resistance traits into crop plants through breeding (Lo Presti et al., 2015).

The lifestyles of plant pathogenic fungi are highly diverse and utilize distinct strategies to interact with the host plant. For example, necrotrophic fungi infect and kill host tissue and extract nutrients from dead host cells. Biotrophic fungi colonize living host tissue and obtain nutrients from living tissue; whereas hemibiotrophic fungi display two phases during the infection process; first is an initial biotrophic phase followed by a necrotrophic stage (Lo Presti et al., 2015). Despite the diversity among these lifestyles, all fungi that colonize plants are recognized by the plant innate immune system, which elicits a host defense response. The innate immune perception triggers both local and systemic reactions, allowing a plant to respond to pathogen attack in a quick and localized manner over an extended period of time (Schwessinger and Ronald, 2012). For this reason, the plant utilizes a two-tier innate immune response that involves a combination of localized plasma membrane and intracellular receptors (Jones and Dangl, 2006; Dodds and Rathjen, 2010; Asai and Shirasu, 2015).

The initial tier of the innate response is generally triggered by molecules essential to the pathogen and cannot be modified without significant loss of viability. These molecules include abundant bacterial proteins, elongation factors Tu (Ef-Tu), flagellin, and fungal cell wall components such as polysaccharides and chitin (Win et al., 2012; Newman et al., 2013). These components are usually exposed and are referred to as pathogen-associated molecular patterns (PAMPs) and microbe-associated molecular patterns (MAMPs). During infection, PAMPs present within the plant apoplast and are recognized by membrane-localized pattern recognition receptors (PRRs), which initiates the first reaction of defense called PAMP-triggered immunity (PTI) (Jones and Dangl, 2006; Koeck et al., 2011). In *Arabidopsis thaliana*, the flagellin receptor, FLS2, a leucine rich repeat (LRR) receptor-like kinase, recognizes the highly conserved N-terminus of bacterial flagellin (flg22) and activates the MAP kinase pathway to induce expression of defense response genes (Gomez-Gomez and Boller, 2002). In addition, changes in hormone biosynthesis and plant cell reinforcement by callose deposition have been shown to occur upon PRR activation (Macho and Zipfel, 2014). Interestingly, oligomers of fungal chitin are recognized by the LysM-RLK (receptor-like kinase) AtCERK1 *A. thaliana* receptor via three extracellular domains (Miya et al., 2007; Liu et al., 2012). The resulting chitin-induced homodimerization of CERK1 has been shown to be essential for the activation of downstream signaling (Liu et al., 2012). In a recent study, however, a lysin motif receptor kinase (LYK) termed AtLYK5 was shown to be the primary chitin receptor, not AtCERK1 (Cao et al., 2014). Interestingly, the AtLYK5 appears to directly interact with AtCERK1, forming a chitin inducible complex to induce plant defense (Cao et al., 2014). Similary in rice, the CEBiP, a LysM-receptor like-protein (RLP), was also shown to directly bind chitin elicitors and interact with OsCERK1, a homolog of AtCERK1, in a chitin-dependent manner (Kaku et al., 2006; Shinya et al., 2012). Studies have shown that reduced expression of either *CEBiP* or rice *CERK1* in RNA interference (RNAi) lines results in an impaired response to chitin elicitors (Kaku et al., 2006; Shimizu et al., 2010). This suggests that both of these molecules are required for chitin signaling in rice.

To successfully facilitate infection, or to establish compatible interactions that lead to proliferation, fungi must be able to counteract PTI. To suppress the immune response and manipulate host cell physiology, plant pathogens secrete effector proteins (Stergiopoulos and de Wit, 2009; de Jonge et al., 2011; Giraldo and Valent, 2013). Although these secreted proteins are key players in suppressing PTI, they are also recognized by the plant surveillance system, which in turn triggers the second tier of immune response termed effector triggered immunity (ETI). Effectors that elicit an ETI response can be recognized by plant resistance proteins (R proteins), which are intracellular nucleotide-binding leucine rich repeat (NLR) receptors (Cui et al., 2015). Recognition of effector proteins via NLR receptors occurs through direct (receptor-mediated binding) or indirect (accessory protein-mediated) interactions (Dodds and Rathjen, 2010; Cui et al., 2015). Activation of ETI results in disease resistance and is usually associated with a hypersensitive cell death response (HR) localized at the infection site (Jones and Dangl, 2006). The strong HR response and resulting phenotype is a product of what is termed host specific gene-for-gene interactions, where an effector, coined Avr (avirulence), is recognized by the cognate R-protein produced by the host plant (Dodds and Rathjen, 2010). To date, ~83 effector proteins have been cloned and characterized from crop-infecting fungi and oomycetes (Table 1); 43 of which are encoded by *Avr* genes. In addition, a majority of cognate plant R-proteins associated with a specific Avr have also been identified (Stergiopoulos and de Wit, 2009; Gururania et al., 2012; Ali et al., 2014). Elucidation of the role of Avr effectors in virulence and the underlying mechanisms involved remains a challenge. Nevertheless, recent research is beginning to reveal the function of increasing numbers of fungal effectors bringing forward new technologies that may help address some of these knowledge gaps and improve our understanding in plant-pathogen interactions.

**Table 1:** **Effectors of well-characterized biotrophic and hemibiotrophic plant pathogenic fungi^**a**^ and oomycetes that have been cloned and studied to date (Excludes toxins)**.

**Effector Protein^b^**	**Length^c^**	**N-terminal signal motif or conserved domains**	**Localization *in planta***	**Expression**	**R-protein association; role in virulence**	**Genomic location**	**References**
***Blumeria graminis f. sp hordei*** **(biotroph; host: barley)**							
AvrA10	286	Unknown	Cytoplasm (predicted)	Unknown	Interaction with Mla10	TE-rich region	Ridout et al., 2006; Shen et al., 2007
AvrK1	177	Unknown	Cytoplasm (predicted)	Unknown	Interaction with Mlk1	TE-rich region	Ridout et al., 2006
*Cladosporium fulvum* (biotroph; host: tomato)							
Avr 2	78	Unknown	Apoplast	*In planta*	Interaction with Cf2. Binds and inhibits tomato cysteine proteases Rcr3 and Pip3	Not reported	Dixon et al., 1996
Avr4	135	Unknown	Apoplast	*In planta*	Interaction with Cf4. Protects against chitinases	Not reported	Joosten et al., 1994; van den Burg et al., 2006
Avr4E	121	Unknown	Apoplast	*In planta*	Interaction with Cf4-E	Not reported	Westerink et al., 2004
Avr9	63	Unknown	Apoplast	*In planta*	Interaction with Cf9	Not reported	van Kan et al., 1991; van den Hooven et al., 2001
**Extracellular proteins (Ecp)**							
Ecp1	65	Unknown	Apoplast	*In planta*	Required for full virulence	Unknown	Lauge et al., 1997
Ecp2	143	Unknown	Apoplast	*In planta*	Required for full virulence	Unknown	Lauge et al., 1997
		Unknown	Apoplast	*In planta*	Unknown	Unknown	Lauge et al., 2000
Ecp5	98	Unknown	Apoplast	*In planta*	Unknown	Unknown	Lauge et al., 2000
Ecp6	199	Unknown	Apoplast	*In planta*	Reduces plant defense response by binding to chitin	Unknown	Bolton et al., 2008
Ecp7	100	Unknown	Apoplast	*In planta*	Unknown	Unknown	Bolton et al., 2008
***Fusarium oxysporum f***. **sp**. ***lycopersici*** **(hemibiotroph; host: tomato)**							
Avr1 (Six4)	242	Unknown	Xylem	Induced during infection	Interaction with I-1	B-chromosome (TE-rich region)	Houterman et al., 2008
Avr2 (Six3)	163	RxLR motif-like	Xylem (translocated to cytoplasm)	Induced during infection	Interaction with I-2	B-chromosome (TE-rich region)	Houterman et al., 2009
Avr3 (Six1)	284	Unknown	Xylem	Stimulated by living cells	Interaction with I-3. Aggressiveness determinant.	B-chromosome (TE-rich region)	Rep et al., 2004
**Secreted in xylem proteins (SIX)**							
Six5	119	Unknown	Xylem	Likely induced during infection	Required for virulence; pairs with Avr2 for full I-2 resistance	Chromosome 14; upstream *Avr2*; shares promoter region	Ma et al., 2015
Six6	199	Unknown	Xylem	Induced during infection	Suppresses I-2 mediated cell death	B-chromosome	Gawehns et al., 2014
***Leptosphaeria maculans*** **(hemibiotroph; host: oilseed rape)**							
AvrLm1	205	Unknown	Cytoplasm (predicted)	Induced during infection	Interaction with Rlm1 and LepR3	TE-rich, AT-Isochore	Gout et al., 2007; Šašek et al., 2012; Larkan et al., 2013
AvrLm2	232	Unknown	Cytoplasm (predicted)	Likely induced during infection	Interaction with Rlm2 (predicted)	TE-rich, AT-isochore; with unique feature harboring a second gene (head-to-head) forming a small GC-isochore	Ghanbarnia et al., 2015
AvrLm3	160	Unknown	Cytoplasm (predicted)	Induced during early infection	Interaction with Rlm3 (predicted)	TE-rich, AT-Isochore	Plissonneau et al., 2016
AvrLm4-7	143	(R/N)(Y/F)(R/S)E(F/W) motif along with RAWG motif located in nearby loop	Possibly apoplastic then translocated into plant cells	Induced during infection	Interaction with Rlm4 and Rlm7; strongly required for virulence;	TE-rich, AT-Isochore	Parlange et al., 2009; Blondeau et al., 2015; Nováková et al., 2015
Avrlm6	144	RxLR-like motif	Cytoplasm (predicted)	Induced during infection	Interaction with Rlm6	TE-rich, AT-Isochore	Fudal et al., 2007, 2009; Kale et al., 2010; Van de Wouw et al., 2010
Avrlm11	95	Unknown	Cytoplasm (predicted)	Induced during infection	Interaction with Rlm11	Dispensable minichromosome;AT-rich isochore	Balesdent et al., 2013
AvrlmJ1 (Avrlm5)	141	Unknown	Cytosplasm (predicted)	Induced during infection (predicted)	Interaction with Rlm5	AT-rich isochore	Van de Wouw et al., 2014, unpublished
***Magnaporthe oryzae*** **(hemibiotroph; host: rice)**							
Avr-Pita 1	224	Unknown	Cytoplasm	*In planta*: accumulate in BIC	Interaction with Pi-ta	Telomeric	Orbach et al., 2000; Khang et al., 2010
PWL1	147	Unknown	Cytoplasm	*In planta:* accumulate in BIC	Unknown	Telomeric	Khang et al., 1995, 2010
PWL2	145	Unknown	Cytoplasm	*In planta:* accumulate in BIC	Unknown	TE-associated	Khang et al., 1995; Sweigard et al., 1995
ACE1	4035	Unknown	Fungal Appresorium	Expressed in appressoria	Encodes a polyketide synthase; Insertion of MINE retrotransposon into ACE1 gene responsible for virulence in strain 2/0/3	*M. oryzae* genome	Bohnert et al., 2004; Fudal et al., 2005
Avr-CO39	89	Unknown	Cytoplasm	Expression *in planta*	Interaction with CO39; Interaction with RGA4/RGA5	TE-associated	Li et al., 2009; Cesari et al., 2013
AvrPiz-t	108	Unknown	Unknown	Unknown	Interaction with Piz-t. Reduces flg-22 and chitin induced ROS generation; Targets U3 ubiquitin ligase from host for degredation for suppressing PTI	TE-associated	Zhou et al., 2006; Li et al., 2009; Park et al., 2012
AvrPia	85	Unknown	Cytoplasm	Expression *in planta*	Interaction with Pi-a	TE-associated	Yoshida et al., 2009
AvrPii	70	C2H2 zinc finger-like motif	Cytoplasm	Expression *in planta*	Interaction with Pi-i	Subtelomeric and TE-associated	Yoshida et al., 2009
AvrPik/km/kp	113	Unknown	Cytoplasm	Expression *in planta*	Interaction with Pi-k	Genome	Yoshida et al., 2009
AvrPib	75	Unknown	Cytoplasm (predicted)	Unknown	Interaction with Pi-b	TE-associated	Zhang et al., 2015
**Secreted LysM protein**							
SLP1	162	LysM domain	Apoplast	Expression *in planta*	Required for full virulence; Binds chitin and suppresses chitin-induced plant immune response	Not reported	Mentlak et al., 2012
MC69	54	No signal motifs identified	Apoplastic	Expression during early infection stage	Required for virulence	Chromosome 6	Saitoh et al., 2012
**Biotrophy-associated secreted (BAS) proteins**							
BAS1	115	Unknown	Cytoplasm	*In planta:* accumulate in BIC	Unknown	Unknown	Mosquera et al., 2009
BAS2	102	Unknown	Cell wall crossing points	*In planta:* accumulate in BIC	Unknown	Unknown	Mosquera et al., 2009
BAS3	113	Unknown	Cell wall crossing points	*In planta:* accumulate in BIC	Unknown	Unknown	Mosquera et al., 2009
BAS4	102	Unknown	BIC	*In planta*	Unknown	Unknown	Mosquera et al., 2009
***Melamspora lini*** **(biotroph; host: flax)**							
AvrL567	150	RxLR-like motif	Cytoplasm	Expressed in haustoria	Interaction with L5, L6, and L7 Not required for full virulence	Unknown	Dodds et al., 2004; Lawrence et al., 2010
AvrM	314	RxLR-like motif	Cytoplasm	Expressed in haustoria	Interaction with M	Unknown	Catanzariti et al., 2006; Kale et al., 2010
AvrP123	117	Unknown	Cytoplasm	Expressed in haustoria	Interaction with P1, P2 and P3	Unknown	Catanzariti et al., 2006
AvrP4	95	Unknown	Cytoplasm	Expressed in haustoria	Interaction with P4	Unknown	Catanzariti et al., 2006
***Puccinia graminis f. sp tritci*** **(biotroph; hosts: wheat and barley)**							
RGDBP	818	RGD motif	Apoplast	Constitutive	Interaction with Rpg1	Unknown	Nirmala et al., 2011
VPS9	744	Forms complex with RGDBP	Apoplast	Constitutive	Interaction with RpgA	Unknown	Nirmala et al., 2011
PGTAUSPE-10-1	Unknown	Unknown	Unknown	Expressed in haustoria	Possible interaction with Sr22	Unknown	Upadhyaya et al., 2014
***Ustilago hordei*** **(biotroph; host: barley)**							
UhAvr1	170	Not reported	Not reported	Not reported	Interaction with Uh1	TE-rich	Grewal et al., 2008
***Ustilago maydis*** **(biotroph; host: maize)**							
Pep1 (protein essential for penetration1)	178	Not reported	Apoplast	Induced during early infection	Required for full virulence; Directly interacts with POX12 and suppresses plant defense by scavenging ROS	Not reported	Doehlemann et al., 2009; Hemetsberger et al., 2012; Hof et al., 2014
Pit2 (protein involved in tumors 2)	118	Not reported	Apoplast	Induced during infection	Required for full virulence; Directly interacts with cysteine protease	Not reported	Doehlemann et al., 2011
Cmu1		Not reported	Apoplast	Induced during infection	Required for full virulence; A chorismate mutase that potentially cooperates with maize chorismate mutase to reduce available SA	Not reported	Eberhard et al., 1996; Djamei et al., 2011
Tin2 (Tumor inducing 2)	207	Not reported	Apoplast	Induced during infection	Required for full virulence; Interacts with maize TKK1 which positively influences anthocyanin production	Not reported	Brefort et al., 2009; Tanaka et al., 2014
See1	157	Not reported	Expressed in nucleus and cytoplasm	Strongly expressed in seedling	Required for full virulence	Not reported	Redkar et al., 2015
***Phytophthora infestans*** **(oomocyte; hosts: potato and tomato)**							
Avr2	118	RxLR motif	Cytoplasm	Induced during infection	Interaction with R2	TE-rich	Lokossou et al., 2009; Gilroy et al., 2011b
Avr3a	147	RxLR motif	Cytoplasm	Induced during infection	Interaction with R3a; INF1-meidated plant death	TE-rich	Armstrong et al., 2005; Bos et al., 2010; Gilroy et al., 2011a
Avr4	287	RxLR motif	Likely cytoplasm	Induced during infection	Interaction with R4	TE-rich	van Poppel et al., 2008, 2009
AvrBlb1; Ipi01	153	RxLR motif	Cytoplasm	Induced during infection	Interaction with RpiBlb1; Disrupts cell wall-plasma membrane adhesion	TE-rich	Pieterse et al., 1994; Senchou et al., 2004; Vleeshouwers et al., 2008
AvrBlb2	101	RxLR motif	Cytoplasm	Induced during infection	Interaction with RpiBlb2; Required for full virulence; blocks plant protease secretion	TE-rich	Oh et al., 2009; Bozkurt et al., 2011
SNE1 (Suppressor of Necrosis1)	291	RxLR motif	Cytoplasm	Induced during infection	Suppresses NLP-triggered and effector-triggered cell death	TE-rich	Kelley et al., 2010
**Cr****inkling and** **n****ecrosis inducing effectors (CRN)**							
PiCRN2	456	Conserved FLAK (for Phe, Leu, Ala, Lys) motif	Cytoplasm	Induced during infection	Triggers plant cell death	TE-rich	Schornack et al., 2010
PiCRN8	599	Conserved FLAK motif	Cytoplasm	Induced during infection	Triggers plant cell death; C-terminal kinase-like domain exhibits kinase activity-suggests role in modification of host-cell signaling pathway	TE-rich	Schornack et al., 2010; Liu et al., 2011
PiCRN15	615	Conserved FLAK motif	Cytoplasm	Induced during infection	Triggers plant cell death	TE-rich	Schornack et al., 2010
PiCRN16	618	Conserved FLAK motif	Cytoplasm	Induced during infection	Triggers plant cell death	TE-rich	Schornack et al., 2010
**Extracellular protease inhibitors (EPI)**							
EPIC1	126	Cystatin-like domain	Apoplast	Induced during infection	Binds and inhibits tomato cysteine proteases Rcr3 and PIP1	Not reported	Tian et al., 2007; Song et al., 2009
EPIC2A	125	Cystatin-like domain	Apoplast	Unknown	Unknown	Not reported	Tian et al., 2007
EPIC2B	125	Cystatin-like domain	Apoplast	Induced during infection	Binds and inhibits tomato cysteine proteases Rcr3 and PIP1	Not reported	Tian et al., 2007; Song et al., 2009
EPIC3	131	Cystatin-like domain	Apoplast	Constitutive	Unknown	Not reported	Tian et al., 2007
EPIC4	173	Cystatin-like domain	Apoplast	Constitutive	Unknown	Not reported	Tian et al., 2007
EPI1	149	Cystatin-like domain	Apoplast	Induced during infection	Binds and inhibits tomato serine protease P69B	Not reported	Tian et al., 2004
EPI10	228	Cystatin-like domain	Apoplast	Induced during infection	Binds and inhibits tomato serine protease P69B	Not reported	Tian et al., 2005
***Phytophthora sojae*** **(oomocyete; host: soybean)**							
Avr1b-1	138	RxLR motif	Cytoplasm	Induced during infection	Interaction with Rps1b and Rps1k; Suppresses plant cell death	TE-rich	Shan et al., 2004; Dou et al., 2008b
Avr1a	122	RxLR motif	Cytoplasm	*In planta*	Interaction with Rps1a	TE-rich	Qutob et al., 2009
Avh331; Avr1k	279	RxLR motif	Cytoplasm	Induced during infection	Interaction with Rpsk; Strongly suppresses PAMP-triggered and effector-triggered cell death	TE-rich	Dou et al., 2008b; Kale et al., 2010
Avr3a/5	111-119	RxLR motif	Cytoplasm	Induced during infection	Interaction with Rps3a and Rps5; Suprresses effector-triggered cell death	TE-rich	Qutob et al., 2009; Dong et al., 2011; Wang et al., 2011
Avr3b	314	RxLR motif	Cytoplasm	Strongly induced in germinating cysts and during infection	Interaction with Rps3b; Suppresses effector-triggered cell death and ROS production	TE-rich	Dong et al., 2011
Avr3c	220	RxLR motif	Cytoplasm	Expressed in early infection stage	Interaction with Rps3c	TE-rich	Dong et al., 2009
Avr4/6	123	RxLR motif	Cytoplasm	Induced during infection	Interaction with Rps4 and Rps6	TE-rich	Dou et al., 2010
Avr1d	125	RxLR motif	Cytoplasm	Induced during infection	Interaction with Rps1d; Suppressed BAX (proapoptotic protein)-induced cell death	TE-rich	Na et al., 2013; Yin et al., 2013
Avh172	218-227	RxLR motif	Cytoplasm	Expressed in early infection stage	Suppresses effector-triggered cell death; Required for virulence	TE-rich	Wang et al., 2011
Avh238	134-142	RxLR motif	Cytoplasm	Induced during infection	Suppresses PAMP-triggered cell death; required for full virulence	TE-rich	Wang et al., 2011
PsCRN63	450	Conserved FLAK motif	Cytoplasm	Constitutive; Slightly induced during infection	Triggers plant cell death	TE-rich	Liu et al., 2011
PsCRN115	449	Conserved FLAK motif	Cytoplasm	Constitutive; Slightly induced during infection	Suppresses plant cell death	TE-rich	Liu et al., 2011
PsNPP1; NLPps	237	Not reported	Apoplast	Induced during transition to necrotrophy	Triggers plant cell death	TE-rich	Qutob et al., 2006

a *Plant pathogenic fungi listed in table are known to infect crop plants (excluded Arabidopsis thaliana)*.

b *Includes proteins that have shown specific host-interaction phenotypes or indication of host cell entry*.

c *The number of amino acid residues within the unprocessed protein*.

In this review we focus on effector molecules of biotrophic and hemibiotrophic fungi, taking a close look at the mechanisms involved in release and uptake of effector molecules by the fungi and plant cells, respectively. We place emphasis on how effectors were discovered, and difficulties associated with determining the effector repertoire. We then discuss the role of specific effectors found in biotrophic and hemibiotrophic fungi and look at how new technology for generating direct mutations may provide a new avenue for elucidating the function of fungal effector proteins.

## Effector delivery mechanisms of fungal pathogens

Effectors can be defined as molecules that alter host cell structure and function, facilitating infection (virulence factors or toxins) and/or triggering defense responses (avirulence factors: Avr). These proteins can be grouped into two classes based on their target sites in the host plant (Kamoun, 2006, 2007). Apoplastic effectors are secreted into the plant apoplast, where they interact with extracellular targets and surface receptors, whereas cytoplasmic effectors are translocated inside the plant cell (Djamei et al., 2011; Dong et al., 2011; Yaeno et al., 2011; Park et al., 2012). Regardless of the effector type, efficient delivery of effectors to the plant is required for the infection process. Since pathogenic fungi have developed distinct lifestyles, they have also established diverse effector delivery systems upon infection. Biotrophic and hemibiotrophic fungal pathogens feed and live on living host cells, and secrete effectors that are targeted for the host apoplast or cytoplasm using specialized infection structures such as appressoria or haustoria (Kemen et al., 2005; Catanzariti et al., 2006; Rafiqi et al., 2010;Koeck et al., 2011; Petre and Kamoun, 2014). Obligate biotrophs such as rust fungi or powdery mildew fungi are only capable of propagating on living host tissue. After penetration of the epidermal cell wall, a lobed haustorium develops within the meshophyll (Manners and Gay, 1983; Mackie et al., 1991; Harrison, 1998). The haustorium is completely surrounded by a membrane termed the extrahaustorial membrane, which appears to be an invagination of the plant plasma membrane (Garnica et al., 2014). Interestingly, the biotroph *Ustilago maydis* does not establish bulbous feeding structures similar to the haustoria-forming rust fungi. During penetration, the host plasma membrane invaginates and completely encloses the intracellular hyphae, establishing an extensive area of interaction where the exchange of molecules between the fungus and host occurs (Djamei and Kahmann, 2012; Lo Presti et al., 2015). Hemibiotrophic pathogens utilize a biotrophic phase early in the infection process followed by a necrotrophic phase killing host cells to complete their lifecycle. For the hemibiotrophic pathogen, *Magnaporthe oryzae*, two discrete secretion systems for delivery of apoplastic and cytoplasmic effectors are utilized (Giraldo et al., 2013). For cytoplasmic effector delivery, these proteins appear to accumulate in the biotrophic interface complex (BIC) near the tip of the first bulbous cell formed after host cell penetration. Apoplastic effectors, on the other hand, are not associated with BIC, and once secreted they are dispersed in the extracellular space between the fungal cell wall and the extra-invasive-hyphal membrane (Giraldo et al., 2013; Zhang and Xu, 2014). There have been no studies to elucidate effector delivery strategies employed by the pathogen *Leptosphaeria maculans*, a hemibiotroph that causes disease in *Brassica* plant species. However, one might speculate that it uses a mechanism similar to that of *M. oryzae*, where some of the cytoplasmic Avr effectors accumulate at the BIC. One particular review suggested that *L. maculans* was an apoplastic pathogen and unlike BIC-forming hemibiotrophs, it triggers a slower defense response where maximum expression of the effectors does not occur until after an initial endophytic growth phase (Stotz et al., 2014).

## Targeting of effectors to host cells

No system analogous to the bacterial secretion system has been identified in fungi. However, one common theme that has emerged in terms of fungal effector secretion is based on host targeting via N-terminal translocation domains, which are found after the general secretory signal peptide. Insights into the effector movement process have come from studies conducted with oomycete pathogens, which employ a similar infection strategy that are used by fungi (Koeck et al., 2011). In the oomycetes, secreted effector proteins share a common N-terminal host targeting domain, which contains common motifs such as RxLR (Arg-x-Leu-Arg), LxLFLAK or CRN (Crinkler motif) and CHxC amino acid sequences (Jiang et al., 2008). The N-terminal RxLR motif along with a downstream DEER (Asp-Glu-Glu-Arg) amino acid sequence in the Avr3a effector protein of *Phytopthera infestans* has been shown to be required for translocation into potato cells (Whisson et al., 2007; Dou et al., 2008a; Bos et al., 2010; Kale et al., 2010).

Identification of N-terminal signal motifs involved in cell entry is not as clearly defined for most fungi as it appears to be for oomycetes. In general, fungal effectors do not appear to share significant sequence similarity; a feature attributed to its rapid variation and host adaptation (Sperschneider et al., 2015a), although some exceptions have been reported. For example, the *Cladosporium fulvum* Ecp6 effector contains a LysM domain, which was shown to align with 16 putative *C. fulvum* Ecp6-like proteins from: *Aspergillus niger, Magnaporthe grisea, Mycosphaerella fijiensis, M. graminicola, Botrytis cinerea, Sclerotinia sclerotiorum, A. nidulans, A. oryzae, A flavus, C. lindemuthianum*, and *L. maculans* (Bolton et al., 2008). Also, a chorismate mutase effector, Cmu1, secreted by the maize pathogen *U. maydis*, illustrated a role in virulence, suggesting that some effectors may, based on protein sequence, have predicted functional roles (Djamei et al., 2011). It should be noted that evidence exists demonstrating conservation within the N-terminal sequence motifs of some fungi. In the barley powdery mildew fungus, *Blumeria gramanis* f. sp. *hordei*, for example, the effector proteins share an N-terminal [YFW]xC motif within 30 amino acids of the signal peptide (Godfrey et al., 2010). This particular motif has also been reported in other effectors found within rust fungi, but amino acid position within the proteins is less conserved (Duplessis et al., 2011). In the genus *Fusarium*, a set of effector proteins share a conserved [SG]PC[KR]P motif located immediately downstream of the N-terminal signal peptide. However, these motifs have yet to be functionally characterized and remain undetermined whether they are indeed fungal effector motifs (Manning et al., 2008). In *Melamspora lini*, the AvrL567 and AvrM effectors enter flax cells autonomously which is governed by N-terminal uptake domains, but the two proteins do not share conserved motifs or structures (Rafiqi et al., 2010).

Other features that may unite groups of effectors include structural similarities, for example the arrangement of secondary structural elements relative to each other, i.e., three-dimensional folds. Studies with the genus *Phytophthora* proteins AVR3a11 and PexRD2 suggested a three-helix bundle fold as the basic structural unit, which is formed by the repeating WY motifs. This particular structural element has been identified in more than 520 related RxLR effector proteins (Boutemy et al., 2011). Interestingly, a duplicated four helical motif with similarity to the WY domain of oomycete effectors was illustrated in the *M. lini* AvrM effector. In a recent study by Goritschnig et al. (2016), the WY-domain of an effector produced by *Hyloperonospora Arabidopsis* (ATR1) was shown to be directly recognized by the *Arabidopsis* NLR RPP1 through association with the C-terminal LRR (Goritschnig et al., 2016), suggesting that this feature may be important for NLR recognition. Other features such as β-sandwich structures have also been discovered in AvrL567 from *M. lini* (Wang et al., 2007) and in the *M. oryzae* effector Piz-t (Zhang et al., 2013), suggesting that structural conservation exists among some fungal effectors. Despite this commonality, a lack of significant effector protein conservation is expected to hamper the use of these features for predicting effector function.

## Identifying and predicting effector candidates

Prior to the advent of genomic sequencing, identification of many effectors was carried out by genetic map-based cloning. Examples include AvrPi-ta (Orbach et al., 2000), ACE1 (Bohnert et al., 2004), and Avr-CO39 (Farman and Leong, 1998) from *M. oryzae*; AvrLm1 (Gout et al., 2006), Avrlm4-7 (Parlange et al., 2009), AvrLm6 (Fudal et al., 2007) from *L. maculans*. The availability of transcriptomic and fungal genomic sequences has accelerated the number of effectors identified in the last decade. By screening for cDNA clones that associate with avirulence loci, Avr3a (Armstrong et al., 2005) and Avr4 (van Poppel et al., 2009) from *P. infestans* and AvrL567 from *M. lini* (Dodds et al., 2004) were identified and characterized. In some cases expressed sequence tags (EST) were screened bioinformatically to identify potential secreted proteins with specific N-terminal secretion signals (Vleeshouwers et al., 2008; Oh et al., 2009; Zhu et al., 2012). Interestingly, analysis of a cDNA library from barley tissue containing the ascomycete *B. graminis* f. sp. *hordei* illustrated that the majority of expressed fungal genes encoded unrelated small proteins containing N-terminal signal peptides and an N-terminal motif of [YFW]xC (Godfrey et al., 2010). Identification of effectors using these types of approaches defined structural properties of effector proteins allowing for bioinformatic predictions of putative effectors. Prediction of hundreds of potential RxLR effector genes in *P. sojae, P. ramorum, P. infestans* (Tyler et al., 2006; Jiang et al., 2008; Haas et al., 2009), *L. maculans* (Rouxel et al., 2011) and powdery mildew fungi were made possible through using a bioinformatic strategy.

Despite the identification of conserved sequence features among effectors, fungal effector prediction approaches remain problematic as they are largely based on relatively broad criteria, which principally rely on the presence of a secretion signal and the fact that the majority of effector proteins are small in size and cysteine-rich (Sperschneider et al., 2015a). These features have been used to mine predicted secretomes, and although it is helpful in reducing the number of candidates, the problem remains that not all secreted small cysteine rich proteins will function as an effector and conversely not all fungal effectors will be small and cysteine-rich. For instance, some cytoplasmic effectors translocated into the host cell are low in cysteine residues and are quite large in size. In *L. maculans*, AvrLm1 effector has only one cysteine residue (Gout et al., 2006), suggesting that the criteria of small and cysteine rich, while being valuable for apoplastic effectors, may have a tendency to exclude potential effector candidates (Sperschneider et al., 2015b).

New approaches for predicting both apoplastic and cytoplasmic effector candidates have been developed that do not rely solely on the presence of a secretion signal, small size and high cysteine residue content. Saunders et al. (2012) designed a thorough *in silico* analysis to identify the potential effector repertoire from the genome of two pathogenic rust fungi. The pipeline is founded on the observation that known effector proteins from filamentous pathogens have at least one of the following properties: (i) the presence of a secretion signal, (ii) being encoded by genes that are induced *in planta*, (iii) exhibiting similarity to haustorial proteins, (iv) small and cysteine rich, (v) the presence of a known effector motif or a nuclear localization signal, (vi) being encoded by genes with long intergenic regions, (vii) the presence of internal repeats, (viii) a lack of PFAM domains, excluding those associated with pathogenicity. The authors then used clustering algorithms to group protein families of rust pathogens and rank them according to the likelihood of being effectors. Using this approach they were able to identify approximately eight families of effector contenders. This pipeline was also utilized in a study conducted on *M. lini*, where 200 potential effector candidates were identified (Nemri et al., 2014), suggesting that this methodology may be useful in future effector predictions. A similar strategy was developed to predict effector candidates in *S. sclerotiorum*. This method involved the equivalent selection pipeline utilized by Saunders et al. (2012), as described above, in addition to selection of proteins belonging to duplicated gene families, and those that illustrated signatures of positive selection (Guyon et al., 2014). With this strategy, the authors were able to putatively identify 78 effector candidates from a predicted proteome of over 14,500 proteins (Guyon et al., 2014), suggesting that the additional criterion for selection within this pipeline could potentially narrow down the number of effector candidates, although more studies need to be carried out to ensure that potential effector proteins are not being missed. Taken together, these studies clearly illustrate that with the development of computational algorithms and enhancement of specific analytical criteria for protein selection pipelines, predicting fungal effectors is becoming more efficient. By looking beyond sequence similarity based methods and integrating relevant physiological effector functions such as gene expression *in planta*, genomic features, or taxonomic information, computational analytical algorithms will prove to be an extremely powerful tool for predicting effector candidates within a large proteome.

Proteomics is another important tool utilized for large-scale studies on proteins that are involved in plant-pathogen interactions. Characterization of a subset of proteins during plant-pathogen interactions has been able to provide a more direct view of cellular processes compared to DNA or RNA analysis and has resulted in the identification of both fungal and oomocyte effectors (Ricci et al., 1989; Kamoun et al., 1993; De Wit et al., 2002; Rose et al., 2002; Rep et al., 2004; Xu et al., 2007; Houterman et al., 2008, 2009; Cao et al., 2009). One of the first studies using proteomics to identify an avirulence gene was carried out by De Wit research group (1986 and 1988) on the pathogenic fungi *C. fulvum*. In these studies, the gene product encoded by *avr9* (Avr9) was characterized through protein purification of tomato apoplastic fluids during compatible/incompatible fungal-plant host interactions using polyacrylamide gel electrophoresis (PAGE), reverse-phase HPLC and EDMAN N-terminal sequencing (De Wit et al., 1986; Schottens-Toma and DeWit, 1988). In *F. oxysporum* f. sp *lycopersici* (FOL), one of the first effectors, termed SIX1 (Avr3), was identified and sequenced using mass spectrometry (MS) (Rep et al., 2004). More specifically, the peptide sequence SIX1 was obtained from purification of xylem sap from infected tomato plants, and a gene deletion was able to further confirm that the absence of the *SIX1* gene resulted in the loss of resistance on an I-3 (corresponding R gene) tomato line. Other studies conducted in the FOL isolate followed suite, where identification of 14 additional SIX proteins were determined through the analysis of the xylem sap proteome of infected tomato plants using two-dimensional gel separation and mass spectrometry (Houterman et al., 2008, 2009; Lievens et al., 2009; Ma et al., 2010; Schmidt et al., 2013). A recent study using a large-scale proteomics approach determined the protein changes in the xylem sap proteome following FOL infection from analyzing plants inoculated with either the wild type or *SIX* knockout FOL isolates (Gawehns et al., 2015). The authors illustrated that the absence of a single effector altered the abundance of the sap xylem proteins, which corresponded to the altered virulence visualized on the plants inoculated with the SIX knockout strains (Gawehns et al., 2015). Clearly, proteomics is an extremely effective tool for finding effector proteins that are expressed during pathogen-host interactions. Even though there are some limitations associated with this type of technique, such as low protein abundance, sample complexity, sensitivity, resolution, and speed of data acquisition (Gonzalez-Fernandez et al., 2010), proteomics appears to be more direct, where proteins that are necessary for plant-pathogen interactions are more precisely identified. This is in contrast to computational prediction systems, which have a tendency to broadly select effector proteins that may or may not have an actual function in plant-pathogen interactions. Furthermore, a more global application can be used in proteomics where the overall contribution of a specific effector on the abundance of proteins within the proteome can be assessed. This type of global analysis will allow for a better understanding of plant-pathogen association and provide insight into the mechanism involved in effector recognition.

## Factors that affect evolutionary changes in effectors

With the development of new computational tools for predicting effectors, researchers have focused their attention on how factors such as genome structure and gene transfer impact fungal effector composition. Fungal effector composition seems to be driven by the evolutionary arms race between effector recognition by plant R-proteins, however where they are located within the genome and how they are transferred from one isolate to another can significantly influence fungal effector repertoire.

Association of the pathogen with its host plant results in either an incompatible (resistant) or a compatible (susceptible) interaction. The fundamental principle explaining the outcome of this interaction is based on the gene-for-gene model (Flor, 1956). This model proposes that the product of the R-gene from the host plant distinguishes an Avr (Avirulent) effector produced by the pathogen leading to a resistant or incompatible reaction. If there is no detection, as a result of allelic variation or lack of one of two components, then a susceptible or compatible reaction is observed. Interestingly, plant-associated microbes continually evolve new effectors to retain or improve their ability to cause disease and to minimize detection by a plant host. Plants are also compelled to maintain disease resistance and improve their ability to detect pathogen effectors by developing new R-gene allelic varations. The antagonistic cycles of evolution between pathogen effectors and plant R-proteins is described as the co-evolutionary “arms-race” or zigzag model (Jones and Dangl, 2006; Dodds and Rathjen, 2010; Tyler and Rouxel, 2013). As a result of this arms race, the genotype for each of these proteins become highly polymorphic and include single-nucleotide polymorphisms (SNPs), insertions and deletions within the gene sequence (Guttman et al., 2014).

An ongoing change in gene alleles especially effector genes, provides plant pathogens with the ability to evade detection while optimizing or maintaining virulence. To ensure survival, pathogens may have to evolve new effectors to successfully colonize and infect a new host target. Adaptation to hosts is believed to be enabled by compartmentalization of effector genes within the genome. Plant pathogenic fungi and oomycetes contain gene-sparse genomic regions that are exceedingly enriched in repetitive elements and putative effector genes (Raffaele and Kamoun, 2012). The movement of transposable elements (TEs) within these genome compartments results in gene duplication, horizontal gene transfer and gene loss, all of which contribute to virulence factor evolution (Lo Presti et al., 2015). For example, the fungal pathogen *L. maculans* has an unusual genome structure compared to other pathogenic fungi in that it contains alternating GC and AT-rich blocks termed GC- and AT-isochores. The GC-isochore regions are enriched with housekeeping genes, whereas the AT-isochores are relatively gene-sparse, enriched in TEs which are truncated and further reduced by repeat-induced point mutations (RIP). The AT-isochore harbors ~122 genes encoding small secreted proteins (Rouxel et al., 2011), which also includes the conditionally dispensable chromosome (Soyer et al., 2014). In *F. oxysporum*, all known effector genes are found in one of its four dispensable chromosomes (Ma et al., 2010) and in *P. infestans* effector genes are located in highly plastic genomic regions, enriched in TEs (Haas et al., 2009).

Horizontal gene transfer (HGT) is another important mechanism by which effector diversification occurs in fungal pathogens. One particular study conducted on *M. oryzae* revealed that the Avr-Pita effector had been translocated several times via mobile elements. The authors suggested that multiple translocations implicate deletions and recoveries mediated via parasexual transfer among individual isolates (Chuma et al., 2011). In addition to intraspecies genetic transfer an HGT event was also reported between different species documented by the transfer of ToxA along with hAT transposase from the wheat blotch pathogen *Stagonospora nodorum* to *Pyrenophora tritci-repentis*, which is the causal agent of tan spot in wheat (Friesen et al., 2006). These studies highlight how selection can influence specific evolutionary changes leading toward diversification of virulence-promoting effectors.

## Function of characterized fungal effectors in biotrophs and hemibiotrophs

Unlike bacterial and oomycete effectors, a limited number of fungal effectors have been functionally characterized. The primary obstacles in fungal effector research derive from difficulties associated with manipulating fungi in a laboratory setting, in particular obligate biotrophs such as rust fungi. Adding to these challenges is the fact that many effector mutants display no associated phenotype, likely due to functional redundancy, poor assay systems or the inability to accurately measure small changes in phenotype. However, technologies such as bimolecular fluorescent complementation (BiFC) (Kerppola, 2008; Kodama and Hu, 2012), immunocolocalization (Dunn et al., 2011) yeast-two hybrid systems (Bruckner et al., 2009) and gene expression assays have significantly advanced our understanding of the functional roles of secreted fungal effectors. In this section, we will discuss the function of biotrophic and hemibiotrophic fungal effectors and emphasize which of the above mentioned techniques were used to further understand *in planta* effector localization, or protein-protein interactions.

### Effectors identified in biotrophs

To date, functional analysis of effectors from biotrophic pathogens including *U. maydis* and *C. fulvum* are among the most extensively studied. Much has been revealed about the effectors in the maize pathogen *U. maydis*. With a relatively small genome of 20.5 Mb, ~50 secreted proteins have been predicted, 50% of which are novel (Kamper et al., 2006; Koeck et al., 2011; Djamei and Kahmann, 2012). Many of these novel genes are localized within gene clusters, and appear to be upregulated during host colonization, and encode effectors that have virulence function (Kamper et al., 2006; Schirawski et al., 2010). As shown in Table 1, five effector proteins, Pep1, Pit2, Cmu1, Tin2, and See1 have been well-characterized.

Pep1 (protein essential for penetration 1) is a small (178 amino acids) secreted effector of *U. maydis* and related smut fungi that has been shown to accumulate in the apoplast. Doehlemann et al. (2009) confirmed the presence of Pep1 within the apoplastic spaces within the leaves, in addition to finding protein accumulation at sites of cell-to-cell passage using *in vivo* immunocolocalization (Doehlemann et al., 2009). Inoculation of leaves with *pep1* deletion mutants showed a failure to establish compatible interactions along with large necrotic patches. Interestingly, the Δ*pep1* mutants were defective for penetration of the initial epidermal cell and cell-to-cell spread. Pep1 has also been shown to be required to overcome extensive host resistance. Using bimolecular fluorescence complementation (BiFC) a direct interaction between the Pep1 protein and the maize defense peroxidase POX12 was established. It appears that Pep1 is able to suppress plant defense mechanisms through scavenging reactive oxygen species (ROS) (Hemetsberger et al., 2012). Interestingly, a recent report further supported the role of Pep1, as it was shown to be necessary for inducing hypersensitive response that displayed necrotic cell death features (Hof et al., 2014).

Pit2 (protein involved in tumors 2) is a secreted, apoplastic effector of *U. maydis* that is required for virulence; studies have shown that a mutation within the *pit2* gene results in attenuation of tumor formation (Doehlemann et al., 2011). A recent study illustrated through the utilization of a yeast-two hybrid system that Pit2 directly interacts with maize cysteine proteases. Using a complementary approach, these researchers illustrated that when the recombinant Pit2 protein was co-inoculated with cysteine protease (CP2), the protease activity was significantly reduced, indicating that Pit2 inhibits the proteases activity via direct interaction. It was also noted that the CP2 protease utilized in the study was recently identified in the leaf apoplasts as an important factor required for Salicylic acid (SA)-associated defense (Mueller et al., 2013), suggesting the importance of Pit2 in virulence.

Cmu1 is an effector that is translocated into the host cell. The *cmu1* gene of *U. maydis* is most strongly induced during plant colonization, leading to an abundance of Cmu1 protein accumulation detected within the apoplast (Djamei et al., 2011). Through complementation analysis in yeast, along with *in vitro* enzymatic assays, Cmu1 was shown to be a chorismate mutase. As the branching metabolite of the shikimate pathway, chorismate is the precursor for synthesis of aromatic amino acids and the plant defense hormone salicylic acid. Data obtained from utilizing a two hybrid system indicated that Cmu1 can form heterodimers with the plastidic as well as the cytosolic forms of plant chorismate mutase (Eberhard et al., 1996; Djamei et al., 2011). Immunocolocalization studies illustrated that Cmu1 was located within the cytosol after translocation into the host plant cell, where the flow of chorismate was redirected through potential cooperation between the cytosolic maize chorismate mutase along with Cmu1, leading to a reduction in available chorismate for salicylic acid biosynthesis (Eberhard et al., 1996). Interestingly, Cmu1 has the ability to spread locally to neighboring yet uninfected host cells (most likely via plasmodesmata), which the authors suggest is a form of metabolic priming leading to lower salicylic acid levels, allowing cells to prepare for impending colonization by *U. maydis* (Eberhard et al., 1996).

The effector Tin2 (Tumor inducing 2) is part of the largest cluster of effectors identified in *U. maydis* and was shown to be responsible for anthocyanin induction during biotrophic phase and have a role in virulence (Brefort et al., 2009). Tin2 localization was shown using confocal microscopy, which illustrated the accumulation of the protein around the fungal hyphae, which is secreted into the apoplast and functions within the cytosol (Tanaka et al., 2014). Inside the plant, the Tin2 protein appears to interact with the protein TTK1 (Tin2-targeting kinase1), a maize protein kinase that regulates the anthocyanin biosynthetic pathway (Tanaka et al., 2014). The interaction between Tin2 and TKK1 was observed using a yeast two-hyrbid screen, where the region of contact between the two proteins coincided with a variable region containing a phosphodegron motif, which is the target for the ubiquitin ligase complex that leads to protein degredation through the ubiquitin-proteasome system (Ravid and Hochstrasser, 2008; Spoel et al., 2009). (Tanaka et al., 2014) illustrated that the Tin2 protein protects the active kinase against ubiquitination as the full length TKK1 was only detectable on SDS-PAGE when co-expressed *in planta* with a functional Tin2 protein. The stabilization of TKK1 via Tin2 appears to positively stimulate the production of anthocyanin in infected tissue and suppress lignin biosynthesis, a common defense pathway (Tanaka et al., 2014).

The *U. maydis* effector protein See1 (Seedling efficient effector 1) is recognized as an organ-specific effector that induces tumor expansion specifically on maize leaves (Redkar et al., 2015). It appears that this protein is expressed in the nucleus and cytoplasm of maize as live cell imaging and immunolabeling using transmission electron microscopy were able to detect the See1-mCherry tagged protein within these two areas of the plant cell (Redkar et al., 2015). See1 is required for reactivation of DNA synthesis, which is an important step for tumor progression in maize leaf cells (Walbot and Skibbe, 2010). Through yeast-two hybrid analysis, it was shown that this protein interacts with maize homolog of SGT1 (Suppressor of G2 allele of *skp1*), a factor that has been shown to act in cell cycle progression in yeast (Dubacq et al., 2002) and an essential component of innate immunity in plant and animals (Shirasu, 2009; Zhang et al., 2010). Further analysis of this interaction illustrated that the See1 protein restricts MAPK-triggered phosphorylation of maize SGT1, which could potentially modulate the immune responses and DNA reactivation synthesis within leaf cells (Redkar et al., 2015).

*C. fulvum* produces at least 10 different effector proteins (Table 1), three of which have been functionally characterized; Avr4, Ecp6, and Avr2. The Avr4 and Ecp6 effectors are involved in suppression of chitin-induced PTI. Avr4 binds to chitin in the fungal cell wall, which protects the fungi against hydrolysis caused by plant chitinases (van den Burg et al., 2006). The Ecp6 effector appears to sequester chitin oligosaccharides, which are released by cell walls during hyphal invasion, allowing the suppression of host immunity (Bolton et al., 2008; de Jonge et al., 2010). A study focused on structural formation of the Ecp6 protein illustrated that two of the three LysM protein domains undergo ligand-induced dimerization, resulting in a high affinity chitin-binding pocket, while the third LysM domain binds to chitin with lower affinity. The authors suggest that binding of this protein out-competes the plant chitin receptor by interfering with its dimerization (Sanchez-Vallet et al., 2013).

The Avr2 effector produced by *C. fulvum* specifically targets secreted cysteine proteases, similar to Cmu1 in *U. maydis*. One particular study using heterologous expression of *Avr2* in *A. thaliana* illustrated an enhanced susceptibility *in planta* toward extracellular fungal pathogens, which included *B. cinerea* and *Verticillium dahlia*. Interestingly, microarray analysis demonstrated that *Avr2* expression generated global changes in the transcriptome reflecting pathogen challenge. Through monitoring protease activity, Avr2 was found to inhibit multiple extracellular Cys proteases, including Rcr3 and its close relative Pip1. In addition, infection by *C. fulvum* was significantly reduced when Avr2 was silenced. Collectively these findings illustrate that Avr2 not only inhibits several Cys proteases required for plant basal defense but it also functions as a virulence factor (van Esse et al., 2008).

### Effectors identified in hemibiotrophs

Hemibiotrophic fungi have a distinct lifestyle wherein they combine a biotrophic phase with a nectrotrophic phase. In this section, we will focus on two hemibiotrophs, *M. oryzae* and *L. maculans*.

A whole genome draft sequence of the *M. oryzae* isolate 70-15 revealed a genome size of 37.8 Mb, with nucleotides encoding ~11,109 proteins (Dean et al., 2005). From the predicted proteome, 1309 genes were predicted to encode secreted proteins (Yoshida et al., 2009). As shown in Table 1, ~16 different effectors have been cloned or characterized to date. Recently, map-based cloning of AvrPib was reported (Zhang et al., 2015), adding another Avr-type effector to the repertoire. MC69 was also shown to be required for pathogenicity; however no evidence was provided to show that this protein suppresses or interferes with plant defense responses (Saitoh et al., 2012). The best characterized effectors to date include SLP1, AvrPiz-t, and ACE1.

SLP1 is a secreted LysM protein that has been shown to be required for *M. oryzae* virulence and is similar to the Ecp6 chitin binding protein found in *C. fulvum*. In a study conducted by Mentlak et al. (2012) live-cell imaging of the GFP-tagged SLP1 illustrated that the protein accumulated at the plant-fungus interface upon rice infection and accrued at the tips of the invading hypae when fungus moved into invading cells (Mentlak et al., 2012). Deletion in the *SLP1* gene reduced the ability of *M. oryzae* to cause disease, which was associated with the inability to proliferate within the host tissues, rather than reducing production of structures required for successful penetration. In a high stringency yeast two-hybrid analysis, SLP1 monomers appeared to associate with one another, suggesting that this protein forms oligomers (Mentlak et al., 2012). The protein was also shown to bind specifically to chitin as purified forms of the protein only co-precipitated with chitin beads or insoluble crab chitin, and not with any other tested polysaccharide (Mentlak et al., 2012). In addition to these findings, the authors determined that SLP1 was able to out-compete the rice PRR chitin elicitor binding protein (CEBiP) for chitin binding when challenged in a competition assay, and suppress chitin-induced oxidative burst,. These findings illustrate that SLP1 binds directly to chitin thereby suppressing chitin-triggered immune response in rice. However, it seems that this function is reliant on the presence of CEBiP, as rice plants silenced with CEBiP RNAi generated disease symptoms when inoculated with the Δ*slp1* mutant (Mentlak et al., 2012). Taken together, the key function of SLP1 as a suppressor of chitin-triggered defense response appears to be reliant on the interaction between SLP1 and CEBiP, which determines the progression of rice blast disease.

AvrPiz-t is a cytoplasmic (Avr-type) effector that has been shown to target the cytosolic rice R-gene (Zhou et al., 2006; Li et al., 2009). AvrPiz-t contributes to disease development in rice when it lacks the corresponding resistance protein Piz-t. Expression of AvrPiz-t in transgenic plant lines illustrated that PTI was suppressed, leading to a reduction in ROS and enhanced susceptibility to *M. oryzae* (Park et al., 2012). A yeast two-hybrid screen identified the interaction between AvrPiz-t with a rice RING E3 ubiquitin ligase APIP6. Ubiquitination of AvrPiz-t by APIP6 was visualized via immune-blotting, and it was shown that after ubiquitination AvrPiz-t-mediated suppression of APIP6 activity occurred (Park et al., 2012).

ACE1, another avirulence gene found in *M. oryzae* has been shown to be upregulated during initiation of infection. ACE1 is not predicted to be secreted or localized to the plant cell cytoplasm, suggesting that the protein itself is not the actual effector but that ACE1 is responsible for the synthesis of an effector that is recognized by the plant R-protein (Bohnert et al., 2004). However, the mechanism by which this occurs has yet to be elucidated.

In *L. maculans*, a total of 122 candidate effectors have been predicted (Rouxel et al., 2011). In terms of Avr effectors, 11 avirulence genes (Avr) termed *AvrLm1, 2, 3, 4, 5, 6, 7, 9, 11, AvrLepR1*, and *AvrLmJ1* have been identified (Balesdent et al., 2002, 2005; Ghanbarnia et al., 2012; Van de Wouw et al., 2014). All of the proteins produced by these genes are known as effectors; small, secreted, cysteine-rich proteins that are non-homologous to any other known *L. maculans* proteins. In plants lacking the corresponding R-protein, the above mentioned Avr effector proteins contribute to disease development; however very little is known about their mechanistic function and whether they are involved in plant immune suppression or virulence specifically. Recently a new study illustrated that the AvrLm4-7 effector was strongly required for virulence (Nováková et al., 2015). Through analysis of plant hormone concentrations, defense gene transcription and ROS accumulation, the authors were able to show that various components of the plant immune system were affected after inoculation with an *L. maculans* isolate lacking a functional AvrLm4-7 allele. Plants inoculated with the isolate carrying a functional AvrLm4-7 gene produced larger cotyledon lesion than the control group isolates that did not harbor an active allele. Interestingly, incompatible interactions due to recognition of AvrLm4-7 by Rlm4 revealed strong early induction of SA and Ethylene (ET)-dependent signaling pathways, which was also previously shown to occur upon recognition of AvrLm1 by Rlm1 (Šašek et al., 2012; Nováková et al., 2015). Through LC-MS/MS chromatography and RT-qPCR analysis (Nováková et al., 2015) determined that an active AvrLm4-7 allele causes suppression of SA-signaling, affecting not only its biosynthesis, but also reducing the expression of the SA-related marker gene *PR1*. The same situation appears to hold true in terms of ET-signaling, where AvrLm4-7 reduces the expression of *ACS2* and *HEL* expression, two ET-responsive genes. These findings together with a recent report illustrating that AvrLm4-7 is translocated into the plant cell (Blondeau et al., 2015) suggest that the plant SA and/or ET hormone signaling pathways, which are critical for induction of the plant defense response (Wang and Irving, 2011), are the primary targets of AvrLm4-7.

## CRISPR/Cas9: a new technique to accelerate our understanding of effector function

From the findings described above, it is apparent that a great deal of progress has been made toward understanding the role of fungal effectors. The most common approach utilized to elucidate the function of these genes involves reverse genetics, where the functional gene is replaced by an antibiotic selection marker, or deleted through homologous recombination. Effector genes in *U. maydis* and *M. oryzae*, as well as pathogenicity genes in *L. maculans* have been successfully deleted using this method (Hemetsberger et al., 2012; Mentlak et al., 2012; Mueller et al., 2013; Feng et al., 2014; Lo Presti et al., 2015). Gene silencing through RNAi has also been used in a number of fungi (Panwar et al., 2013; Petre et al., 2014; Soyer et al., 2014; Whigham et al., 2015; Yin et al., 2015). Although these methods have proven extremely useful, it has become apparent that there is an increasing need in effector research for techniques that can address effector gene redundancy and function within a large protein family. For studies such as these, we are currently limited in terms of fungal selection markers, where it becomes increasingly difficult to generate strains carrying multiple mutations within defined sites. One particular method that is used to recycle antibiotic markers is called the FLP-FRT recombination technique. FLP is a site-specific recombinase, which catalyzes recombination between two directly oriented FRT sites, leading to excision of the intervening DNA segment. Because the antibiotic selection marker is located on the intervening DNA segment, the end result is an unmarked gene disruption (Wirth et al., 2007). In *U. maydis*, this approach represents one of the best ways to introduce multiple mutations within the genome without utilizing all available selection markers; however a number of retro-transformation cycles followed by growth cycles to stimulate the loss of the marker are often required (Khrunyk et al., 2010).

The clustered regularly interspaced short palindromic repeat (CRISPR)-Cas9 system was originally identified in bacteria and archaea as a defense mechanism to prevent the invasion of foreign DNA from phage or plasmids (Barrangou et al., 2007). The CRISPR/Cas9 system has been adapted for use in a number of genome editing applications in both eukaryotes and prokaryotes (Jinek et al., 2012; Cong et al., 2013; Jiang et al., 2013; Doudna and Charpentier, 2014; Nissim et al., 2014; Sander and Joung, 2014). The adapted system consists of a Cas9 nuclease, which is guided to a specific target site by a single guide RNA (sgRNA). The nuclease then generates a double stranded break (DSB) at the desired target site within the genome. The DSB within the DNA initiates the repair mechanism (non-homologous end joining pathway) resulting in short deletions or substitutions, which may cause frameshifts or generate premature stop codons within the ORF of the target DNA (Doudna and Charpentier, 2014). Application of the CRISPR/Cas9 system depends on the expression of the *cas9* gene from *Streptococcus pyogenes* and the ability of the sgRNA to express and fold into the secondary structure necessary for interaction with Cas9 nuclease inside the nucleus.

CRISPR/Cas9-based technologies have emerged as an effective way to generate unmarked mutations in several organisms (Sander and Joung, 2014), including *Saccharomyces cereviseae* (DiCarlo et al., 2013), *Trichoderma reesei* (Liu et al., 2015), *M. oryzae* (Arazoe et al., 2015), and *Neurospora crassa* (Matsu-ura et al., 2015). One particular group was able to show that CRISPR/Cas9 could be modified to enable disruption and replacement of an effector gene in the oomycete *P. sojae* (Fang and Tyler, 2015). In each of the mutations the authors examined, short indels were located specifically at the Cas9 cleavage site, and deletions of one, three and six base-pairs were observed within the target regions providing evidence regarding the effectiveness of this system. Nodvig and colleagues developed a CRISPR/Cas9 system that allows RNA-guided mutagenesis by transforming a target fungus with a single plasmid. This approach was further modified allowing enhanced functionality in a broader range of filamentous fungi through the use of strong promoters from *Aspergillus niger* for *cas9* and sgRNA expression (Nodvig et al., 2015). More intriguingly, the CRISPR/Cas9 system has high efficiency in targeting multiple unrelated genes using several sgRNA, as shown in the yeast *Saccharomyces cerevisiae* (Cong et al., 2013), the filamentous fungus *T. reesei* (Liu et al., 2015) and two plant systems, namely rice and *Arabidopsis* (Endo et al., 2015; Zhang et al., 2015).

Clearly, the CRISPR/Cas9 “craze” has led to a plethora of useful tools that target not only single genes, but multiple gene targets, which will be extremely useful for effector research. The most valuable attribute of the CRISPR/Cas9 systems is its versatility, where the tools can be used to specifically target genes for mutagenesis in many organisms. Certainly, this system will transform and improve biological research, leading to many new discoveries in gene regulation and function. Nevertheless, this system is still in its infancy and as such several aspects need to be refined, including off-targeting and expression of the components within the desired host.

## Conclusion: future challenges

We have gained a wealth of knowledge toward understanding plant-pathogen interactions over the past several decades. Research has shown that effector proteins play a significant role in the ability of fungal pathogens to establish a compatible interaction with the host plant, and in some cases have confirmed their involvement as virulence factors. We have working methods for developing mutations within single genes, and with the introduction of CRISPR/Cas9 technology, we now have the capability to target multiple genes without the use of markers. This system will significantly improve our ability to target redundant or related effector genes at one time, perhaps providing a definitive or visual phenotype that will provide insight into effector function. Despite our improved knowledge in this area, some important factors remain unresolved. Many fungal effectors have been shown to function primarily within the plant cytosol, yet lack translocation signals within the primary amino acid sequence. This brings into question how effectors are targeted to and taken across the plasma membrane of the plant cell? Although some host targeting sequence motifs have been identified in oomycetes, most fungi do not share this common feature. Moreover, the low sequence homologies among these fungal proteins make it difficult to predict similar features. However, the rapid development of next-generation sequencing tools and new bioinformatics algorithms and pipelines will provide new whole-genome information for both biotrophic and hemibiotrophic fungi. Given the large numbers of effector candidates, research will be a challenge. However, with the continuous development of new tools and techniques we will undoubtedly move toward exceptional opportunities for investigating the roles of fungal pathogen effectors in plant-pathogen interactions.

## Author contributions

Concept of article by WD. Also went through all research papers and finalized the review. CS did the undertaking of researching on the topic and writing the review. TD and MB read the manuscript and gave input on improvements.

### Conflict of interest statement

The authors declare that the research was conducted in the absence of any commercial or financial relationships that could be construed as a potential conflict of interest.
